# Rapid evolution of colistin resistance in a bioreactor model of infection of *Klebsiella pneumoniae*

**DOI:** 10.1038/s42003-024-06378-0

**Published:** 2024-07-01

**Authors:** Juan-Carlos Jiménez-Castellanos, Bartlomiej Waclaw, Alison Meynert, Sean P. McAteer, Thamarai Schneiders

**Affiliations:** 1 Chemical Biology of Antibiotics, Centre for Infection & Immunity (CIIL), Pasteur Institute, INSERM U1019—CNRS UMR 9017, Lille, France; 2 School of Physics and Astronomy, The University of Edinburgh, JCMB, Edinburgh, UK; 3 Dioscuri Centre for Physics and Chemistry of Bacteria, Institute of Physical Chemistry, Warsaw, Poland; 4 MRC Human Genetics Unit, MRC Institute of Genetics and Cancer, The University of Edinburgh, Western General Hospital, Edinburgh, UK; 5 Department of Bacteriology, The Roslin Institute and R(D) SVS, The University of Edinburgh, Easter Bush Campus, Midlothian, Edinburgh, UK; 6 Centre for Inflammation Research, Institute of Regeneration and Repair, Edinburgh Medical School, The University of Edinburgh, Edinburgh, UK

**Keywords:** Microbiology, Medical research

## Abstract

Colistin remains an important antibiotic for the therapeutic management of drug-resistant *Klebsiella pneumoniae*. Despite the numerous reports of colistin resistance in clinical strains, it remains unclear exactly when and how different mutational events arise resulting in reduced colistin susceptibility. Using a bioreactor model of infection, we modelled the emergence of colistin resistance in a susceptible isolate of *K. pneumoniae*. Genotypic, phenotypic and mathematical analyses of the antibiotic-challenged and un-challenged population indicates that after an initial decline, the population recovers within 24 h due to a small number of “founder cells” which have single point mutations mainly in the regulatory genes encoding *crrB* and *pmrB* that when mutated results in up to 100-fold reduction in colistin susceptibility. Our work underlines the rapid development of colistin resistance during treatment or exposure of susceptible *K. pneumoniae* infections having implications for the use of cationic antimicrobial peptides as a monotherapy.

## Abstract (web summary)

A combined microbiological and mathematical approach to study colistin resistance in antibiotic sensitive *K. pneumoniae* reveals a rapid emergence and selection of genetic variants generated by spontaneous mutations in key regulatory genes.

## Introduction

*Klebsiella pneumoniae* is an opportunistic highly antibiotic resistant Gram-negative pathogen, currently recognised by CDC and WHO as a severe threat to both human and animal health^1–3^. The emergence of pan-drug-resistant variants of *K. pneumoniae* significantly limit treatment options, demanding an increased reliance on last-resort agents such as tigecycline, carbapenems, and colistin^4,5^.

Colistin, also known as polymyxin E, is a cationic amphipathic polypeptide commonly used to treat drug-resistant Gram-negative bacterial infections. The extensive use of colistin against a wide range of bacterial infections is a testament to its rapid bactericidal activity, which was curtailed following cases of human neuro and nephrotoxicity^6,7^. Despite this, colistin is now used against multiple drug-resistant isolates of Enterobacterales and is usually used in combination with aminoglycosides, tigecycline, and carbapenems^8^.

Colistin has been used in the veterinary context since the 1950s both in the treatment of pro- and meta-phylactic infections, where usage data suggests that it was the 5th most used antimicrobial in food producing animals in Europe in 2011^9^. However, its usage is not limited to the EU but extends globally resulting in the emergence of colistin-resistant isolates of veterinary and environmental origin^10^.

Colistin electrostatically interacts with the anionic bacterial outer membrane, displacing calcium and magnesium ions from lipopolysaccharides (LPS). As such, colistin resistance in Gram-negative bacteria, including *K. pneumoniae*^11,12^ arises through alterations in intrinsic loci encoding two component regulatory systems or via the acquisition of the *mcr*-type plasmid-mediated phosphoethanol amine lipid transferases^13^. In *E. coli* and *Salmonella*, resistance arises due to genetic alterations and subsequent overexpression of the PhoPQ and PmrAB systems which activates the *pmrHFIJKLM operon*. Similarly, in *Klebsiella pneumoniae*, both the PhoPQ, PmrAB and the *Klebsiella*-specific CrrAB loci regulate the *pmrHFIJKLM* (also known as *arnBCADTEF*) locus which alter the negative charge of LPS via the addition of a 4-amino-4-deoxy-L-arabinose moiety to lipid A, thereby limiting colistin binding.

Different studies of colistin-resistant *K. pneumoniae* isolates show that bacteria can harbour multiple mutations which include loss-of-function changes in the small lipoprotein MgrB, a repressor of the PhoPQ system, as well as single point mutations within histidine-kinases (e.g., PhoQ, PmrB and CrrB)^2,14^ leading to the constitutive phosphorylation of the cognate response regulators (i.e., PhoP, PmrA and CrrA)^15–17^ and the subsequent activation of the *pmrHFIJKLM* operon^2,14^. In addition, the activation of the *pmrHFIJKLM* operon has also been linked to the production of connector proteins such as PmrD or CrrC, which trigger either the PmrAB or CrrAB systems, further amplifying the activation of *pmrHFIJKLM* operon and LPS modifications^6^.

Given the multi-mechanistic basis of colistin resistance in *K. pneumoniae*, we sought to understand which de novo chromosomal mutation(s) would arise under treatment scenarios, including colistin^18^. Using a continuous culture bioreactor, to simulate colistin treatment of a *K. pneumoniae* infection^19^ and in combination with whole-genome sequencing, susceptibility testing, and mathematical modelling, enabled us to gain insights into the process of de novo resistance emergence and selection of resistant mutations, which would be difficult to study in human or animal models.

We show that *K. pneumoniae* develops genetic resistance to colistin within 24 h at a clinically relevant concentration of the antibiotic (10 mg L^−1^) based on published standard pharmacokinetic and pharmacodynamic parameters used for colistin regimens^18,20–22^. Our results show that resistance emerges mostly through mutations within the CrrAB and PmrAB loci but not in the lipoprotein MgrB. Over time, we observed some mutations being replaced by better-adapted variants. Our mathematical approach, which models the population dynamics of resistant mutants, reproduces the experimental data and provides insights into the experimentally inaccessible early stages of mutant emergence and growth. We find that colistin selects for the fastest-growing mutants from a large pool of resistant variants which exist at low numbers even before colistin exposure. Further application of our mathematical model shows that colistin resistance is likely to evolve at different bacterial loads and growth rates relevant for real diseases. Together, our approach suggests that intrinsic de novo mutations conferring clinically relevant colistin resistance arise easily in patients during treatment and underscores the importance of using colistin as part of combination therapy.

## Results

### Colistin selects for highly resistant mutants in a single step

**Fig. 1:**
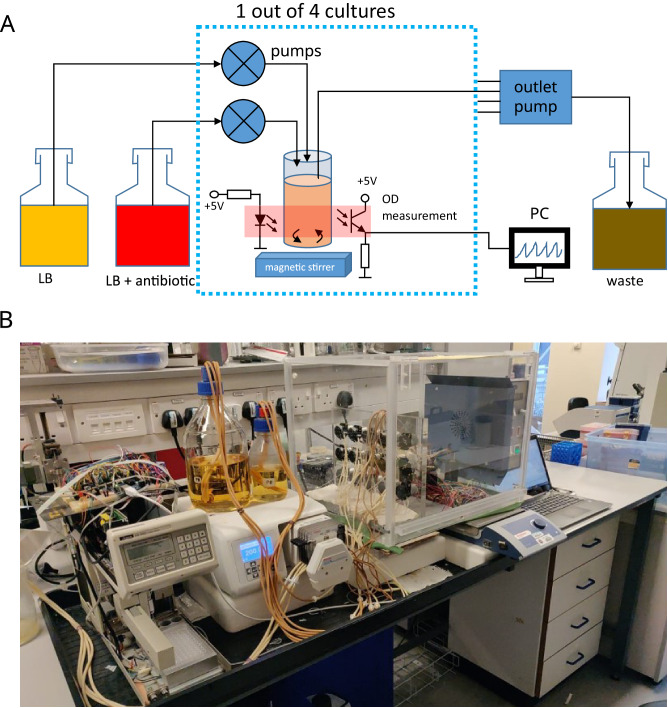
Automated culture system. **A** Schematic diagram. The dotted blue box represents one of four bioreactors; all kept inside an incubator set to 37 °C. Media and waste bottles are outside the incubator. **B** A photograph of the actual system.

We cultured *K. pneumoniae* Ecl8 in an automated bioreactor in which the bacterial culture was kept in a state of continuous growth by periodically diluting it with fresh LB broth (Fig. 1). One hour after the culture first reached the optical density OD = 0.5, colistin was added at the desired concentration (10 mg L^−1^) and maintained at this concentration for the duration of the experiment.

**Fig. 2:**
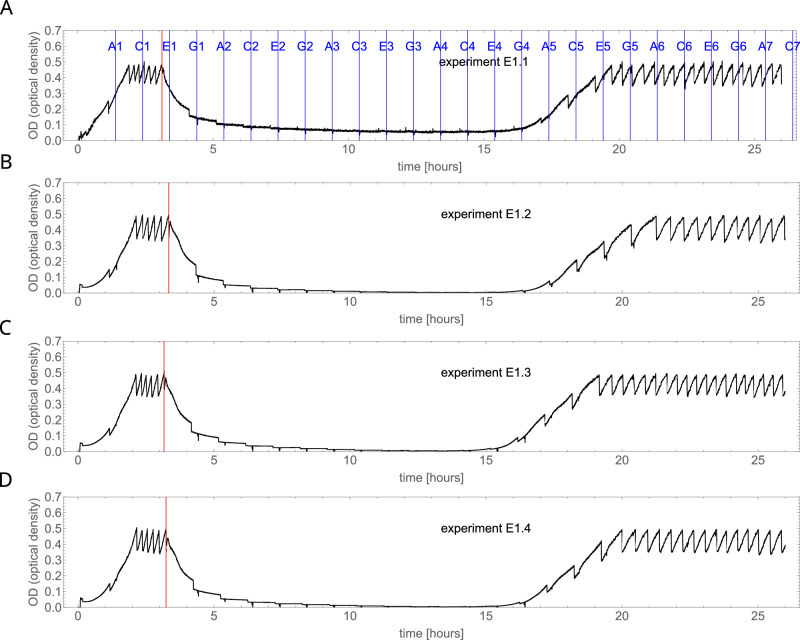
Representative examples of OD vs time curves for four experiments. Red line indicates the beginning of colistin challenge. Blue lines show the times at which the culture was taken by the autosampler, with sample labels denoted A1, C1, …etc. The different panels (**A**–**D**) indicate independent experiments. We performed WGS for samples C5 and E6 in experiment E1.1.

Upon the addition of colistin, cell density sharply decreased below the detection threshold in all cultures within 7 h (Fig. 2A–D). Nevertheless, all cultures eventually recovered pre-colistin challenge cell densities 12–17 h post-colistin exposure. The time from exposure to regrowth showed little variation (Supplementary Information, Fig. S1, mean = 18.3 h, std. deviation = 0.9 h). Cells from the endpoint of the experiment were highly resistant to colistin, with MICs of at least 64 mg L^−1^ and over 256 mg L^−1^ for some cultures (Table 1). Such a significant increase in resistance has been previously reported for *P. aeruginosa* exposed to colistin in the morbidostat; however, resistant variants took 10–20 days to evolve^23^. Importantly, the observed behaviour is not specific to Ecl8 as the same consistent regrowth is observed for *K. pneumoniae* 52145^24^ (Supplementary Information, Fig. S2). Interestingly, *E. coli* laboratory strain MG1655 which lacks the *crrA, crrB* and *crrC* genes, does not evolve resistance under these conditions (Supplementary Information, Fig. S3).

**Table 1:** Colistin susceptibility, mutations and frequency of appearance

Sample	Colistin MIC (mg/L)	Resistant mutations	Mutation frequency
Ecl8 WT	1	No mutations, putative pre-existing pool.	
P1.1*	64	None	
P1.2*	64	CrrB P151L	0.13
		CrrB P151Q	0.32
		PmrB S85R	0.17
		MgrB Q30*	0.07
P1.3*	64	CrrB P151L	0.23
		CrrB P151Q	0.28
		PmrB S85R	0.15
		MgrB Q30*	0.06
P2.1*	64	None	
P2.2*	64	PhoQ G385S	0.24
		PhoQ L26Q	0.08
		CrrB P151S	0.12
		CrrB P151L	0.28
		MgrB W6*	0.12
P2.3*	64	PhoQ G385S	0.22
		PhoQ L26Q	0.03
		CrrB P151S	0.08
		CrrB P151L	0.49
		MgrB W6*	0.04
E1.1*	128	FimD P43R	1
E1.2	128	PmrB S85R (1463686G→T)	0.65
E1.3	128	PmrB S85R (1463688T→G)	0.33
E2.1*	$\ge$256	FimD P43R	1
E2.2	128	CrrB G183V	0.72
E2.3	$\ge$256		
E3.1*	128	PmrB S85R	0.98
E3.2*	128		
E3.3*	64		
E4.1	256	PmrB D150Y	0.9
E4.2*	256		
E4.3	256		
E5.1	$\ge$256	FimD P43R	1
E5.2	256	PmrB S85R (1463688T→G)	0.68
E5.3	256	PmrB S85R (1463686G→T)	0.29
E6.1	$\ge$256	CrrB G183V	0.79
E6.2	$\ge$256	CrrB Q10L	0.12
E6.3	$\ge$256		
E7.1	128	FimD P43R	1
E7.2	256	CrrB G183V	0.85
E7.3	128		
E8.1	128	CrrB S195N	0.82
E8.2	128		
E8.3	128		

MICs of end-point samples, and mutations detected in these samples, gene name, frequency. Asterisk “*” denotes “dry” colonies (large, flat colonies). “Mutation frequency” is a dimensionless quantity between zero and one equal to the fraction of the alternative (mutated) allele in the sample.

### Genetic basis of colistin resistance

**Fig. 3:**
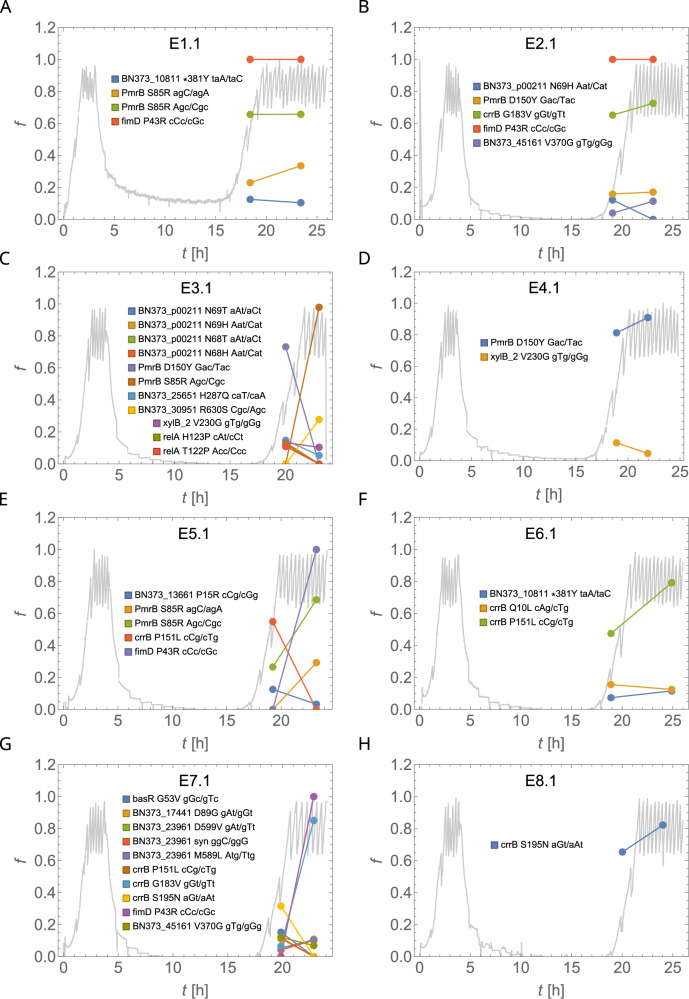
Fraction of alternative alleles (mutations) as a function of time, overlaid with the optical density curves. $f$ is the allele frequency, $t$ is time since the start of the experiment. Grey curve represents OD(t). Colours represent different mutations; their positions and gene names (if known, NCBI GenPept CDS locus tag if not) are listed next to each plot. Panels **A**–**H** reflect the alleles observed at the different time points in independent samples. Non-synonymous-amino acid substitutions have been also shown. “*” denotes a stop gained/stop lost type of mutation.

We detected mutations in genes associated with colistin resistance in all experiments E1.1–E8.1 (Fig. 3A–H). We did not detect any mutations associated with resistance in pre-colistin samples from pilot experiments P1.1 and P2.1 (Supplementary Information, Fig. S4). Thus, we surmise that almost all cells in the cultures tested were initially sensitive to colistin and de novo resistant variants were present in less than 1% of cells (detection limit of our WGS). We classified mutations as common (allele frequency $f \, > \, 0.2$) or rare ($f \, < \,0.2$, i.e., present in less than 20% of sequenced alleles). Common mutations were observed in four genes: *pmrB*, *crrB*, *fimD*, and the hypothetical gene (BN373_30951) (AA changing mutation at 3242969, R630S). All these mutations, except *fimD* and BN373_30951, are known to increase resistance to colistin^25,26^. The mutation P43R in *fimD*, involved in the export and assembly of *fimA* fimbrial subunits across the outer membrane, were clonal (frequency $f\approx 1$) at time point 3 in E1.1 (Fig. 3A), E2.1 (Fig. 3B), E5.1 (Fig. 3E), E7.1 (Fig. 3G) (4 out of 8 WGS samples) and subclonal at time point 2 in E5.1 and E7.1.

Rare mutations were found in many other genes (Supplementary Data Sheet 1). The majority of these genes have not been previously associated with resistance. A small fraction of these genes may be relevant although the evidence for these links rarely extends beyond sequencing analyses and thus are mechanistically weak (Supplementary Information, Table S1). We also found rare *pmrB*, *crrB*, *phoP*, *phoQ* variants where the low frequency was likely caused by late occurrence or lower fitness of these mutations (Table 1).

Due to the limited number of WGS samples ($n=8$ for the main experiment and $n=2$ for the pilot experiments), some resistance mutations may not have been detected in our experiment. Based on the multiplicities of the observed putative resistance mutations, we estimated the number of possible but undetected mutations to be less than 12 (95% Bayesian credible interval), and most likely as few as two (Supplementary Information, SI.3).

### Population dynamics of mutations and their relative fitness

**Fig. 4:**
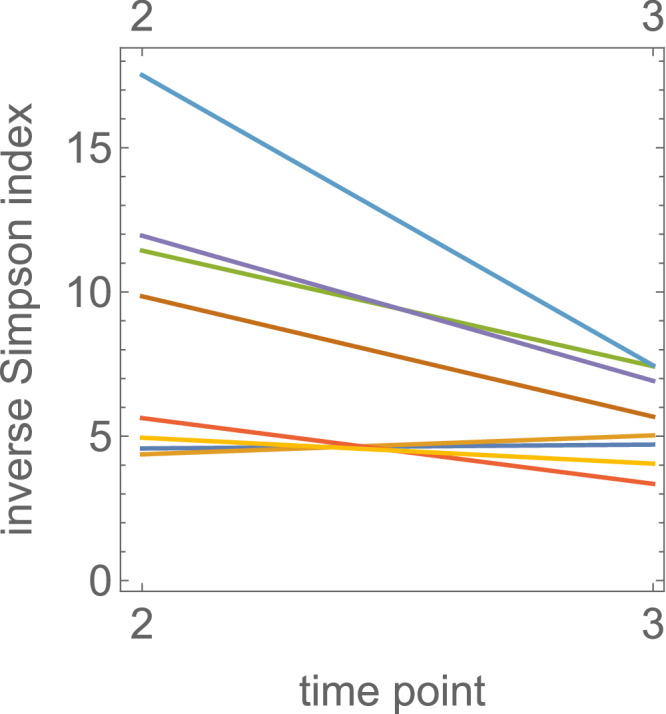
Genetic diversity decreases in time. Inverse Simpson index $S=1/{\sum }_{i}{f}_{i}^{2}$ calculated using alternative allele (mutation) frequencies $\{{f}_{i}\}$ for time points 2 and 3. The numeric value of the index is approximately equal to the number of different alleles (mutations) in the population. The observed decay of $S$ over time suggests selection acting on a set of alleles existing before time point 2.

We observed reduced genetic polymorphisms at time point 3 relative to time point 2 (Fig. 4), which suggests that purifying selection acts against less-fit variants in the presence of colistin. For instance, two mutations in *pmrB* (S85R, D150Y), occurred in 4 out of 8 sequenced experiments. Bioinformatics analysis using PROVEAN^27^ predicted that both mutations should be detrimental in the absence of colistin (Supplementary Information, SI.4). Interestingly, *pmrB* D150Y occurred in E3.1 (Fig. 3C) and E4.1 (Fig. 3D) as the most frequent mutation at time point 2, which was replaced at time point 3 by *pmrB* S85R in E3.1. To check if the clones harbouring mutation S85R in *pmrB* have a higher growth rate than D150Y in the presence of 10 mg L^−1^ colistin, we reconstructed the S85R and D150Y mutations in the *pmrB* gene in *K. pneumoniae* Ecl8 and measured their growth rate with and without colistin (10 mg L^−1^). We found that the relative fitness of the PmrB variants compared to the wild type *K. pneumoniae* Ecl8 are 0.95 + /−0.01 (Ecl8PmrB^S85R^) and 0.97 + /−0.01 (Ecl8PmrB^D150Y^), which is consistent with the PROVEAN predictions (Supplementary Information, SI.4). However, the relative fitness of S85R to D150Y at 10 mg L^−1^ colistin is 0.99 + /−0.02 i.e., consistent with no fitness difference. Thus, the apparent higher growth rate of the S85R-harbouring clone in the bioreactor could be due to the mutation co-occurring with another, fitness-increasing mutation. However, we did not find any evidence of this in the data, as this would require other mutations to have frequencies similar to that of S85R. Thus, we hypothesise that a non-genetic alteration might have occurred in the clone harbouring the S85R mutation (e.g., changes in gene expression)^28^. Variations in *pmrB* (S85R), which arose as a consequence of substitutions at either 253 A→C or 255 C→A, co-occurred with *fimD* P43R in E1.1 (Fig. 3A) and E5.1 (Fig. 3E). In both experiments, its frequency was close to $f=1$, indicating that *pmrB* S85R and *fimD* P43R were present in the same clone. In E5.1, this combination replaced *crrB* P151L between time points 2 and 3, either due to the combination *pmrB* S85R + *fimD* P43R being more fit than *crrB* P151L at 10 mg L^−1^ colistin, or due to non-genetic-adaptation. We observed three frequent ($f \, > \,0.2$) mutations in *crrB* namely, G183V, P151L, S195N all of which have been reported in colistin-resistant *K. pneumoniae*^2,25,26^. A combination of *crrB* G183V and *fimD* P43R outcompeted *crrB* S195N. Interestingly, *fimD* P43R always co-occurred with mutations in *pmrB* and *crrB*. In fact, PROVEAN analysis suggests that the P43R change in *fimD* is deleterious on its own (score −8.23) (see Supplementary Information, SI.4). We hypothesize that this mutation increases the growth rate of resistant clones in the presence of colistin but only in combination with *pmrB* and *crrB* mutations. In previous work, Cain et al.^29^ report that CrrB can function as a regulator of fimbriae formation via *fimD*. However, our data show no differences in MICs in those clones which harboured *pmrB* and *crrB* mutations either singly or in combination with *fimD* (Table 1) suggesting that the *fimD* mutation does not contribute directly to colistin susceptibility.

We also observed several mutations in *mgrB* (a missense variation, a stop gained, and a frameshift variation), albeit at very low frequencies: 2–5% at time point 2, 0–2% at time point 3 (E1.1–E8.1) (Fig. 3A–H) and 4–6% (P1.1 and P2.1) (Supplementary Information Fig. S4 and Supplementary Data Sheet 1). Such a low prevalence of *mgrB* mutations agrees with the results of Cain et al.^29^ and Janssen et al.^16^, who did not observe any *mgrB* mutants in liquid- and plate-based colistin selection experiments^29,30^ of *K. pneumoniae*.

### Resistant mutants emerge before colistin treatment

Resistant cells which cause regrowth in our experiments could arise either before or during colistin challenge. In the case of pre-existing resistance, alternative allele frequency would have to be lower than $\sim$0.01 (<1%); otherwise WGS from time point 1 in experiments P1.1, P2.1 would have revealed the presence of such alleles in the population. To check if a small number of resistant mutants were present in the culture just before colistin exposure, we spot-plated 10 µl of the culture onto LB agar with 10 mg L^−1^ of colistin. Each sample produced a few colonies, corresponding to $\sim {10}^{4}$ resistant cells per culture, suggesting that resistant variants exist in *K. pneumoniae* population before colistin challenge.

### The mathematical model shows resistance is due to a small number of “founder cells”

**Fig. 5:**
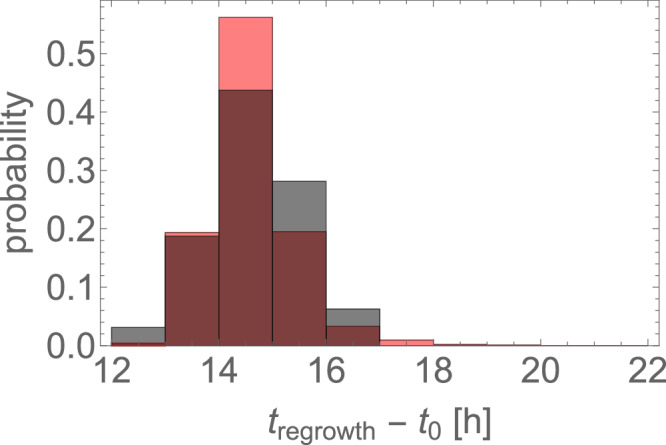
The model reproduces the histogram of regrowth times. Red = simulations with the best-fit mutation probability $\mu =9\times {10}^{-10}$, grey = experimental data.

**Fig. 6:**
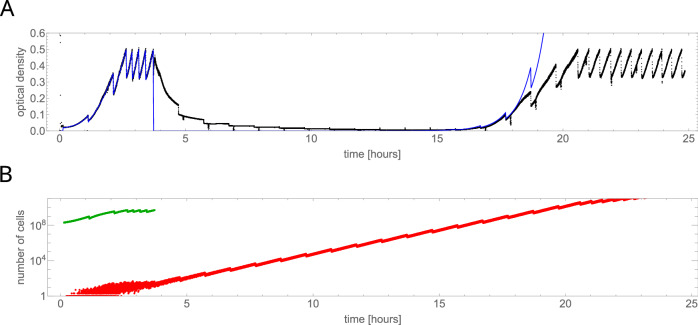
Dynamics of resistant mutants predicted by the computer model. **A** An example simulation run fitted to the OD versus time curve from the experiment E4.2 (black, blue = model). **B** Number of live cells predicted by the model: green = colistin-sensitive, red = colistin-resistant. Different red curves correspond to different realisations of the computer simulation.

We then used a mathematical model to verify our hypotheses about the origin of resistance (“Methods”). To obtain the unknown mutation rate, $\mu$, we fitted the model to the observed distribution of regrowth times from E1.1–E8.1 (Fig. 3A–H) and obtained $\mu =\left(0.6\ldots 1.2\right)\times {10}^{-9}$ (Kolmogorov–Smirnoff test, *P* value > 0.01). Our model reproduces the experimental histogram accurately (Fig. 5). Dividing the estimated $\mu$ by the observed number of putative resistant mutations (2 in *pmrB*, 3 in *crrB*, 1 in *fimD*, and 1 in BN373_30951 gene) from WGS, we obtained an estimate of the per-base, per-replication mutation rate of *K. pneumoniae* as ${\gamma }_{{Kp}}=\left(1\ldots 2\right)\times {10}^{-10}$. We are not aware of any direct measurement of ${\gamma }_{{Kp}}$. However, *K. pneumoniae* mutates with approximately the same rate as *E. coli* in the fluctuation test^31,32^. Our estimate of ${\gamma }_{{Kp}}$ is indeed similar to ${\gamma }_{E.{coli}}\sim 2\times {10}^{-10}$ from *E. coli* mutation accumulation experiments^33^. Assuming that our estimate of ${\gamma }_{{Kp}}$ is correct, the eight resistant mutations detected at the endpoint of the experiment must be a small fraction of all resistant mutations generated before colistin exposure. In particular, at least 26-point mutations in *mgrB* are known to cause resistance^34,35^. This should contribute at least $\approx 26\times 1\times {10}^{-10} \, \approx \, 2.6\times {10}^{-9}$ to the mutation rate $\mu$, twice more than our upper estimate of $\mu$ based on the regrowth time. Some of these mutations might go undetected due to the small number of sequenced samples (Supplementary Information, SI.3). We postulate that *mgrB* mutations do occur in our experiment, but they are rapidly outcompeted by more fit variants with mutations in other genes. This does not require *mgrB* mutations to confer lower fitness in the absence of colistin^12^ but only to be less fit at 10 mg L^−1^ colistin; a similar behaviour has been reported for *E. coli* and fluoroquinolones^36^. Similarly, additional mutations in *pmrB*, *crrB*, and possibly other genes could theoretically occur but did not show up in WGS due to having a lower growth rate. This is supported by our observation that many more resistant cells are present in the culture just before colistin challenge, compared with $\sim 10-50$ “founder cells” predicted by the model (Fig. 6a, b). Moreover, if we assume that there are six distinct resistant mutations, the model predicts on average ${m}_{ > 50 \% }\sim 0.7$ resistant mutations present in >50% of the cells, which is comparable to the actual number $\sim 1$ from the experiment (we exclude *fimD* and BN373_30951 since their role in colistin resistance is unclear). If the number of founder cells was much higher (either more distinct resistant mutations possible, or ${\gamma }_{{Kp}}$ larger than assumed), ${m}_{ > 50 \% }$ would be much lower (Supplementary Fig. S5).

### Resistance evolution is likely in the clinical setting

**Fig. 7:**
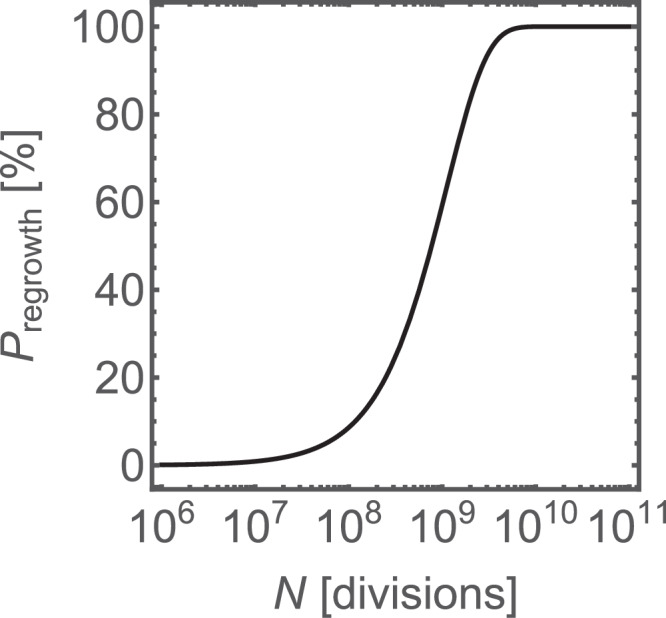
Model extrapolation to different bacterial densities. The probability of evolving de novo resistance versus the number of divisions of *Klebsiella pneumoniae*, for $\mu =0.9\times {10}^{-9}$.

We can now extrapolate the results from our bioreactor to a real infection. The only relevant parameters that affect the probability ${P}_{{{{{{\rm{resistance}}}}}}}$ of resistance evolution are the effective mutation rate $\mu$ at a clinically relevant concentration of colistin used here, and the number of divisions $N$ of sensitive cells that have occurred before colistin treatment. Figure 7 shows ${P}_{{{\rm{resistance}}}}$ as a function of $N$, for the mutation rate $\mu =0.9\times {10}^{-9}$. The probability is close to zero for $N < {10}^{7}$, but approaches 100% for $N > {10}^{9}$. The number of divisions can be estimated from the bacterial load $L$, doubling time ${t}_{d}$, and the duration of infection $T$ before colistin is administered as $N\approx \frac{{LT}}{{t}_{d}}$. This assumes that all resistant mutants survive. If only a fraction of mutants survive, the probability of resistance will be proportionally smaller. We estimate (Supplementary Information, SI.5 and Supplementary Table S3) that $N$ is between ${10}^{3}$ and ${10}^{14}$, depending on the infection type, with numbers 10^5^ to 10^8^ typical for bacteraemia, and $N > {10}^{8}$ for lung infections^37^. Figure 7 shows that, at the high end of the bacterial load spectrum, resistance of *K. pneumoniae* to colistin is certain to evolve under these experimental conditions.

## Discussion

We used a bioreactor model of *K. pneumoniae* infection to better understand the origin and dynamics of resistance evolution to colistin. Although bioreactor models lack the complex host response of animal models, they benefit from higher reproducibility, better control and ability to monitor the de novo emergence of resistance^38^. Compared to earlier work on AMR evolution in bioreactors^23,39^, agarose plates^29^, and batch cultures^16,30,40^, our approach (i) used clinically relevant combinations of pathogen and antibiotic concentrations, (ii) frequent bacterial density measurements, (iii) quantitative mathematical modelling, (iv) fully automated sampling at multiple time points, and (v) multiple replicates. Moreover, the number of cells present in the bioreactor (>10^10^) before colistin challenge ensures a constant supply of mutations. Evolutionary bottlenecks^41^ are less relevant in this setup, and even small fitness differences can lead to large differences in the final frequency of competing alleles.

Our challenge experiments reveal rapid evolution of resistance, with MICs often exceeding 256 mg L^−1^ despite challenge at only 10 mg L^−1^ colistin. Our data are consistent with what has been shown previously in vivo using a mouse peritonitis model where colistin-resistant variants can arise within 24 h from a sensitive strain^28^. WGS shows that the evolved population is multiclonal but that in the presence of colistin the frequency of some alleles increase at the expense of other alleles. However, based on our results we hypothesise that non-genetic adaptations may co-occur with genetic changes to further increase fitness against colistin challenge. Mathematical modelling reproduces the population dynamics of resistant mutants and shows that regrowth is derived a small sub-population of resistant variants existing within a predominantly sensitive population. Less-fit variants, including mutations in *mgrB*, are outcompeted by mutations in *crrB*, *pmrB* which are more fit in these experimental conditions.

Consistent with the broader literature^1,5,42^, our challenge experiments demonstrate that despite the very low frequency of *mgrB* other variants linked to colistin resistance emerge in both the *crrB* and *pmrB* genes. The low frequency of *mgrB* mutations may appear contrary to published studies which show that it is a mutation predominantly found in colistin-resistant clinical isolates associated with the clonal expansion of specific epidemic lineages such as ST258, ST11 and ST15 lineages^4,17,24^. However, our data are in line with other studies of plate or broth-based colistin challenge, which did not detect *mgrB* mutants^17,29^. In fact, recent work suggests that *mgrB* mutations exert a fitness cost consistent with the observed low frequency of isolation in vivo^43^.

Given that 10 mg L^−1^ colistin used reflects a humanised dose^4,18,19,38^ and considering that the ratio between therapeutic concentrations and the MIC is in the order of 5–10 (as much larger concentrations are toxic to the patient)^44^, our study suggests that de novo mutations which confer colistin resistance are likely to occur in the population of *K. pneumoniae* during treatment. Importantly, our data suggests that a single, spontaneously generated mutation in an intrinsic locus can increase the MIC 100× or more in a non-strain-dependent manner supporting the rationale against using colistin and other antimicrobial peptides as a monotherapy for susceptible or resistant *K. pneumoniae* infections.

## Methods

### Bioreactor as a model of *K. pneumoniae* infection

We cultured *K. pneumoniae* Ecl8^44,45^ in an automated bioreactor, similar in construction to the turbidostat^46^ and the morbidostat^47,48^. The system consists of four identical bioreactors. In each bioreactor, bacteria were incubated in a cylindrical glass bottle (~25 mm internal diameter, ~40 mL max. volume) equipped with a magnetic rod driven from underneath by a multi-position stirrer (SciQuip GyroStir 10), a set of inlet and outlet tubes, and an optical sensor for turbidity measurements (Fig. 1). A set of miniature peristaltic pumps connected to media reservoirs delivered the media (LB and 50 mg L^−1^ colistin sulphate (Sigma-Aldrich) in LB). A 4-channel peristaltic pump pumped out spent medium and the surplus bacterial culture. The outlet tube was positioned so that, with the outlet pump running, any surplus volume over the 25 mL culture volume would be removed. Further details are provided in the supplementary information (Supplementary Information, SI.1).

An Arduino microcontroller connected to a PC, running a custom-written (C + +) software to control the experiment, recorded the OD (every 2.5 s) and the temperature (every 25 s). The software initiated dilution with fresh medium every 1 h or when the optical density exceeded OD_600_ = 0.5 or whichever happened first. To dilute, an appropriate volume of fresh media (LB only, or LB + colistin) was first added. Next, the outlet pump ran for 23 s, long enough to pump out any surplus culture and reduce the total volume to 25 mL. The volume of the injected fresh media was such that a fraction $f=0.66$ of the bacterial culture remained in the system (or, conversely, a fraction $1-f=0.34$ was removed).

An automated sampler connected to culture bottle 1 retrieved two 200 µL samples of bacterial culture every hour and injected them into a 96-well plate with empty wells (sample 1), and wells with 100 µL 70% ethanol (sample 2), all covered with transparent sterile seals to prevent evaporation. To ensure that colistin remained stable over a 24-h period, we undertook High-Performance Liquid Chromatography (HPLC) analysis as previously described^49–52^. Briefly, HPLC injection was performed on a C18 XBridge column (5.0 µm, 150 by 2.1 mm [inner diameter]). The mobile phase was 0.1% (vol/vol) trifluoroacetic acid (TFA) in 0.1% acetonitrile (Solvent A) and TFA in water (20:80, vol/vol; Solvent B). The flow rate was 0.4 mL/min with an injection volume of 30 µL. Our protocol generated retention times of mixed polymyxins (internal standard)^52^, colistin A and colistin B of ∼5.8, 7 and 7.2 min, respectively. The *m/z* of mixed polymyxins, colistin A and colistin B were 578, 1155 and 1169, respectively. Since the mixture of polymyxins are stable but too low in abundance to be detected by mass we therefore used UV as described before^49^ for the quantitation of colistin. The bioavailability results are a percentage of mUA change between time=0 h and time = 24 h. All values are the mean of at least three independent biological repeats (Supplementary Information, Fig. S6).

### Evolution of resistance to colistin

We used the colistin-sensitive strain *K. pneumoniae* Ecl8 where the sequenced genome confirms that there are no pre-existing mutations located within intrinsic colistin resistance conferring genes^45^. This is consistent with previous work which shows that *K. pneumoniae* Ecl8 is susceptible to colistin and other antimicrobial peptides and produces no aberrant transcriptional response in relation to intrinsic loci relevant to colistin resistance when grown in LB^29^. We performed eight experimental runs, E1–E8, with 4 independent cultures in each (total 32 biological replicates E1.1–E8.1). After inoculation with 200 µL of dense overnight culture, cells were incubated in the bioreactor without the antibiotic until the OD_600_ reached 0.5 for the first time, and for an hour afterwards. During the next dilution cycle, an appropriate volume of 50 mg L^−1^ colistin sulphate (Sigma-Aldrich) was injected to obtain the desired concentration (10 mg L^−1^) in the culture. This antibiotic concentration was maintained for the rest of the experiment by adding appropriate volumes of LB broth and colistin. The experiment was stopped between 24 and 26 h (for different runs) post antibiotic challenge. We initially performed two pilot runs (P1.1, P2.1) to optimise the experimental protocol. The main difference of these runs to E1–E8 was the increased sensitivity of the optical density detectors, which caused saturation as the OD approached 0.5 resulting in lower OD values being reported than the true OD.

### Whole-genome sequencing

We performed WGS for 8 experimental runs E1.1, E2.1, E3.1, E4.1, E5.1, E6.1, E7.1, E8.1 (Fig. 3A–H), at two time points: a cycle in which OD increased above zero for the first time during the regrowth phase and a cycle close to the end of the experiment. In addition, we sequenced samples from the two pilot experiments (P1.1, P2.1) at three time points: the last pre-colistin cycle, early regrowth following challenge, and near the end. We also sequenced an overnight culture (OVC) grown in LB without colistin to use as the reference genome. Genomic DNA was extracted using Lucigen Kit as per the manufacturer’s instructions (Bioresearch, Lucigen, UK), and samples were sequenced by Novagene (Cambridge, UK) using an Illumina Novaseq platform with 150 bp paired-end reads generating 1GB of raw data (mean coverage 263×). Samples were aligned to *K. pneumoniae* Ecl8 reference genome^45^. Allele variants were called, and their frequencies were obtained as described in SI.2 Section “WGS/Data Analysis”. Supplementary Data Sheet 1 lists all amino acid changing variants and their frequencies.

#### Minimum inhibitory concentration (MIC)

End-point samples from experiments P1.1, P2.1 and E1.1–E8.1 were plated onto colistin agar plates (10 mg L^−1^). From each plate, three different colonies were picked to establish colistin susceptibility using the microbroth dilution method^53^. Briefly, bacterial suspensions (OD_600_ = 0.01) were added to doubling dilutions of colistin (concentration range 0.25–256 mg L^−1^) in LB. Plates were incubated overnight at 37 °C for up to 20 h and absorbance (OD_600_) was read using a Fluostar Optima (BMG Labtech) plate reader. The protocol and breakpoints were based on CLSI guidelines^53^. We note that antibiotic testing guidelines for colistin suggest the use of cation-adjusted Mueller–Hinton broth. However, our tests and other published studies^29,54,55^ show that susceptibilities generated in LB broth are comparable to those in cation-adjusted Mueller–Hinton broth. Importantly, using LB allowed us to establish the susceptibility profiles relevant to the conditions within the bioreactor.

### Generation of genetic variants in *K. pneumoniae* Ecl8

To generate the specific PmrB variants (S85R and D150Y), we first replaced the PmrAB locus with a kanamycin resistance cassette using the gene replacement strategy described for *K. pneumoniae* Ecl8^56^. We amplified 1-kb flanks and the PmrAB operon using (No-Co primers) prior to cloning into pTOF25 replacement vector. This recombinant plasmid was then subjected to site-directed mutagenesis to generate the S85R and D150Y variations. The site-directed mutagenesis was performed according to the manufacturer’s instructions using primers c255gF/R and g488t/c450t F/R (Supplementary Information, Table S4). The variant S85R and D150Y constructs were confirmed using Sanger sequencing prior to electroporation and chromosomal exchange into *K. pneumoniae* Ecl8 *pmrAB*<km > . Successful chromosomal exchanges were verified by the loss of the kanamycin resistance and confirmed using PCR with the Next-Cext primers. Mutants are available upon request, once relevant permissions are in place.

### Mathematical model

We used a model based on the birth-death process with mutations to simulate the population of bacteria in the bioreactor. At time $t=0$ only colistin-sensitive cells were present. Sensitive cells replicated with rate ${r}_{S}\left(t\right)\left(1-\mu \right)$, and mutated into resistant cells with rate ${r}_{S}\left(t\right)\mu$, where $\mu \ll 1$ represented the per-cell per-generation probability of a colistin-resistant mutation. In the absence of colistin, resistant cells also grew with rate ${r}_{S}\left(t\right)$. In the presence of colistin, sensitive cells did not grow, but resistant cells replicated with time-independent rate ${r}_{R}$. Both sensitive and resistant populations were periodically diluted (as in the experiment) by removing a fraction $1-f=0.34$ of all cells.

${r}_{S}\left(t\right)$ was time-dependent to account for changes in the growth rate necessary to reproduce the observed OD vs time curves from before colistin exposure (Fig. 6). Specifically, ${r}_{S}\left(t\right)$ was a piecewise function, each interval representing a period between two consecutive dilutions. To obtain it, we fitted the function $C+\exp \left(A+{Bt}\right)$ to the ${{{{{\rm{OD}}}}}}\left(t\right)$ in each dilution interval and calculated the number of sensitive cells ${N}_{S}={VX}({{{{{\rm{C}}}}}}+\exp \left(A+{Bt}\right))$). Here *V* = 25 ml and $X={4\times 10}^{8}$ ml^−1^ is the proportionality factor representing the density of cells at OD = 1, extrapolated from CFU counts of suspensions of *K. pneumoniae* Ecl8 of OD = 0.1 and OD = 0.5. We then determined the growth rate as ${r}_{S}\left(t\right)\equiv d({{{{\mathrm{ln}}}}}{N}_{S}(t))/{dt}=B(\exp (A+{Bt}))/(C+\exp (A+{Bt}))$.

${r}_{R}$ was assumed to be time-independent since we only simulated the model until the population recovered; OD was low in this phase of the experiments, and resistant bacteria were expected to grow exponentially with constant rate. ${r}_{R}$ was determined as the maximum growth rate of the population following colistin exposure. In all simulations we used ${r}_{R}=1.7\,{{{{{{\rm{h}}}}}}}^{-1}$ which was the average regrowth rate in E1–E8 (Supplementary Information, Fig. S7).

The probability of mutation, $\mu$, was obtained by finding $\mu$ such that the similarity (measured as the *P* value of the Kolmogorov–Smirnoff test) between the experimental and simulated distribution of regrowth times was maximised.

### Relative fitness of PmrB variants S85R and D150Y

We used a previously published method to generate the PmrB variants^56^. Briefly, we incubated bacteria in LB at 37 °C in a 96-well micro-plate (200 μl/well). We started from two different initial cell densities, ${N}_{0}$ in rows A–D and ${N}_{0}/10$ in rows E-H. The ${N}_{0}$ dilution was prepared by 5× dilution of an exponentially growing bacterial culture of the optical density OD_600_ approx. 0.1. We used two columns of the 96-well plate for each mutant: one with no colistin, and another one with 10 mg L^−1^ colistin. We measured the optical density (OD_600_) of each culture every 2 min to obtain growth curve data over a 20 h incubation period using a plate reader (BMG LABTECH FLUOstar Optima). The exponential growth rate was determined from the time shift (time taken for the different bacterial dilutions to achieve the same OD) between the growth curves for initial bacterial concentrations ${N}_{0}$ and ${N}_{0}/10$ as previously explained^57^. The experiment was repeated thrice (three biological replicates in 96-well plates run on three consecutive days), with reordering the position of the wild type and mutants in the plate to control for alterations in growth rate due to temperature variations within the plate reader and uneven evaporation in the plate.

### Statistics and reproducibility

As stated above, we repeated the bioreactor experiment eight times, plus two additional pilot runs. Each run yielded four independent experiments, for a total of $n=8\times 4+2\times 4=40$ experiments. WGS was performed for ten of these experiments. We also performed one run (four experiments) using *E. coli* MG1655 instead of *K. pneumoniae*. Variations between the experiments was inherent to the stochastic nature of evolution of resistance as discussed in “Results”. Antibiotic susceptibility determination was repeated three times for each sample.

### Supplementary information

Supplementary Information

Description of Additional Supplementary Materials

Supplementary DataSheet 1

## Data Availability

The code used to simulate the mathematical model (C + +), a Mathematica notebook that processes the plate reader data, a Mathematica notebook that generates all plots, and all source data including the raw mass spectrometry data are available from Edinburgh DataShare repository (https://datashare.ed.ac.uk/handle/10283/8712). See the file “*A brief description of the dataset*” therein for more information. In particular, to generate all plots, download and run “*Wolfram Mathematica notebook that creates all figures*” along with the required data sets. All genomic data was deposited under accession number PRJEB57212. The source data behind the mutations identified in the whole-genome sequencing can be found in Supplemental Data Sheet 1.
