# Supplementary material for: Rapid evolution of colistin resistance in a bioreactor model of infection of Klebsiella pneumoniae
Source: Commun Biol. 2024 Jul 1;7:794. doi: 10.1038/s42003-024-06378-0 (PMC11217424; doi:10.1038/s42003-024-06378-0)
Supplement: Supplementary file 2 — Description of Additional Supplementary Materials [file 42003_2024_6378_MOESM2_ESM.docx]

**Description of Additional Supplementary Files**

**File name:** Supplementary Data 1

**Description:** contains the information around WGS data
